# The Highly Prolific Phenotype of Lacaune Sheep Is Associated with an Ectopic Expression of the *B4GALNT2* Gene within the Ovary

**DOI:** 10.1371/journal.pgen.1003809

**Published:** 2013-09-26

**Authors:** Laurence Drouilhet, Camille Mansanet, Julien Sarry, Kamila Tabet, Philippe Bardou, Florent Woloszyn, Jérome Lluch, Grégoire Harichaux, Catherine Viguié, Danielle Monniaux, Loys Bodin, Philippe Mulsant, Stéphane Fabre

**Affiliations:** 1 INRA-ENVT, UMR 444, Laboratoire de Génétique Cellulaire, Castanet-Tolosan, France; 2 INRA UMR 85, CNRS UMR 7247, Université de Tours, IFCE, Physiologie de la Reproduction et des Comportements, Nouzilly, France; 3 INRA, SIGENAE, Laboratoire de Génétique Cellulaire, Castanet-Tolosan, France; 4 INRA, GeT-PlaGe Genotoul, Castanet-Tolosan, France; 5 INRA, Plate-forme d'Analyse Intégrative des Biomolécules, Laboratoire de Spectrométrie de Masse, Nouzilly, France; 6 UMR 1331 INRA-ENVT-EIP-INPT-UPS, Toxicologie Alimentaire, Toulouse, France; 7 INRA, UR 631, Station d'Amélioration Génétique des Animaux, Castanet-Tolosan, France; University of Bern, Switzerland

## Abstract

Prolific sheep have proven to be a valuable model to identify genes and mutations implicated in female fertility. In the Lacaune sheep breed, large variation in litter size is genetically determined by the segregation of a fecundity major gene influencing ovulation rate, named *FecL* and its prolific allele *FecL^L^*. Our previous work localized *FecL* on sheep chromosome 11 within a locus of 1.1 Mb encompassing 20 genes. With the aim to identify the *FecL* gene, we developed a high throughput sequencing strategy of long-range PCR fragments spanning the locus of *FecL^L^* carrier and non-carrier ewes. Resulting informative markers defined a new 194.6 kb minimal interval. The reduced *FecL* locus contained only two genes, insulin-like growth factor 2 mRNA binding protein 1 (*IGF2BP1*) and beta-1,4-N-acetyl-galactosaminyl transferase 2 (*B4GALNT2*), and we identified two SNP in complete linkage disequilibrium with *FecL^L^*. *B4GALNT2* appeared as the best positional and expressional candidate for *FecL*, since it showed an ectopic expression in the ovarian follicles of *FecL^L^*/*FecL^L^* ewes at mRNA and protein levels. In *FecL^L^* carrier ewes only, B4GALNT2 transferase activity was localized in granulosa cells and specifically glycosylated proteins were detected in granulosa cell extracts and follicular fluids. The identification of these glycoproteins by mass spectrometry revealed at least 10 proteins, including inhibin alpha and betaA subunits, as potential targets of B4GALNT2 activity. Specific ovarian protein glycosylation by B4GALNT2 is proposed as a new mechanism of ovulation rate regulation in sheep, and could contribute to open new fields of investigation to understand female infertility pathogenesis.

## Author Summary

Prolific sheep have proven to be a valuable model to identify genes and mutations implicated in ovarian function and female fertility. Indeed, fecundity genes of the Bone Morphogenetic Protein (BMP) family discovered in sheep was evidenced as genetic candidates to explain female infertility pathologies. Studying French Lacaune sheep breed, we discovered another fecundity gene named *B4GALNT2*, encoding a glycosylation enzyme that is not related to the BMP family. The high prolificacy of Lacaune sheep was explained by overexpression of B4GALNT2 in the ovary leading to atypical glycosylation of inhibin, an important hormone regulating ovarian function. Our findings open promising fields of investigation to better understand female fertility disorders.

## Introduction

Women, cattle, goats and ewes have generally one or two offspring, whereas other mammals, such as sows, rodents and dogs are prolific and produce more than three offspring. It relies on the number of ovulations at each estrus cycle i.e. the ovulation rate (OR), for which the underlying genetic mechanism was puzzling until the identification of fecundity genes in sheep, bone morphogenetic protein-15 (*BMP15*), growth and differentiation factor-9 (*GDF9*) and BMP receptor-1B (*BMPR1B*) [1]. Following the discovery of sheep fecundity genes, several research groups have focused on *BMP15* and *GDF9* and they have found numerous mutations associated with human ovarian pathologies such as premature ovarian failure or polycystic ovary syndrome [2]. Thus, prolific sheep are now considered as valuable models for identifying genes and mutations involved in mechanisms controlling the ovarian function, for agronomical purposes such as genetic selection of prolificacy, and for clinical purposes in the case of female infertility or subfertility.

In the meat strain of the Lacaune sheep breed, large variation in litter size has been observed and genetic studies explained this variation by the segregation of at least two major genes influencing OR and prolificacy, one being X-linked and named *FecX*, the second being autosomal and named *FecL* [3], [4]. *FecX* is known as *BMP15* and in the Lacaune breed, the mutant allele (*FecX^L^*) associated with high prolificacy was identified as a pCys321Tyr substitution altering the BMP15 protein function [3]. Heterozygous *FecX^L^* mutation is associated with a twofold increase in OR, but homozygous *FecX^L^*/*FecX^L^* ewes are sterile, thus mimicking the phenotype observed for the other 5 mutations described in the ovine *BMP15* gene [5]–[7].

The influence of the autosomal *FecL^L^* mutation on OR is additive with one copy increasing OR by about 1.5 and two copies by about 3.0 [4], [8]. We have recently established that the *FecL* locus influences both the ovarian activity and the endocrine profiles [9]. Indeed, increased OR in homozygous *FecL^L^*/*FecL^L^* (thereafter named *L/L*) ewes is associated with an increased number of gonadotropin-dependent follicles with a diameter greater than 3 mm, an increase in plasma estradiol concentrations, and an increase in the frequency of Luteinizing Hormone (LH) pulsatility during the follicular phase, leading to a precocious LH surge. In contrast, plasma concentrations of Follicle Stimulating Hormone (FSH) were not different compared to wild-type ewes. Based on ovarian phenotype and endocrine profiles, these findings suggest that the *FecL^L^* mutation affects ovarian function in a different way compared to other known hyperprolificacy-associated mutations, all affecting genes of the bone morphogenetic protein signaling system, *BMP15*, *GDF9* and *BMPR1B* [1], [10].

The *FecL^L^* mutation associated with increased OR has not yet been identified. In a previous work, a full genome scan localized the *FecL* locus on sheep chromosome 11 (OAR11). Fine mapping reduced the interval containing *FecL* to markers BM17132 and FAM117A, corresponding to a synteny block of 1.1 megabases on human chromosome 17 (HSA17), which encompasses 20 genes [8]. With the aim to identify the *FecL* gene and its hyperprolificacy-associated mutation, we combined different approaches based on genetic fine mapping (both classical development of genetic markers and high throughput Roche 454 sequencing strategy), gene expression analysis, histochemistry and protein identification by mass spectrometry. From our results, we propose *B4GALNT2*, encoding the glycosylation enzyme beta-1,4-N-acetyl-galactosaminyl transferase 2, as the *FecL* gene. Finally, the *FecL* sheep model of prolificacy-associated mutation leads to the discovery of a new pathway involved in the regulation of folliculogenesis and ovulation rate.

## Results

### Fine mapping

**Figure 1:**
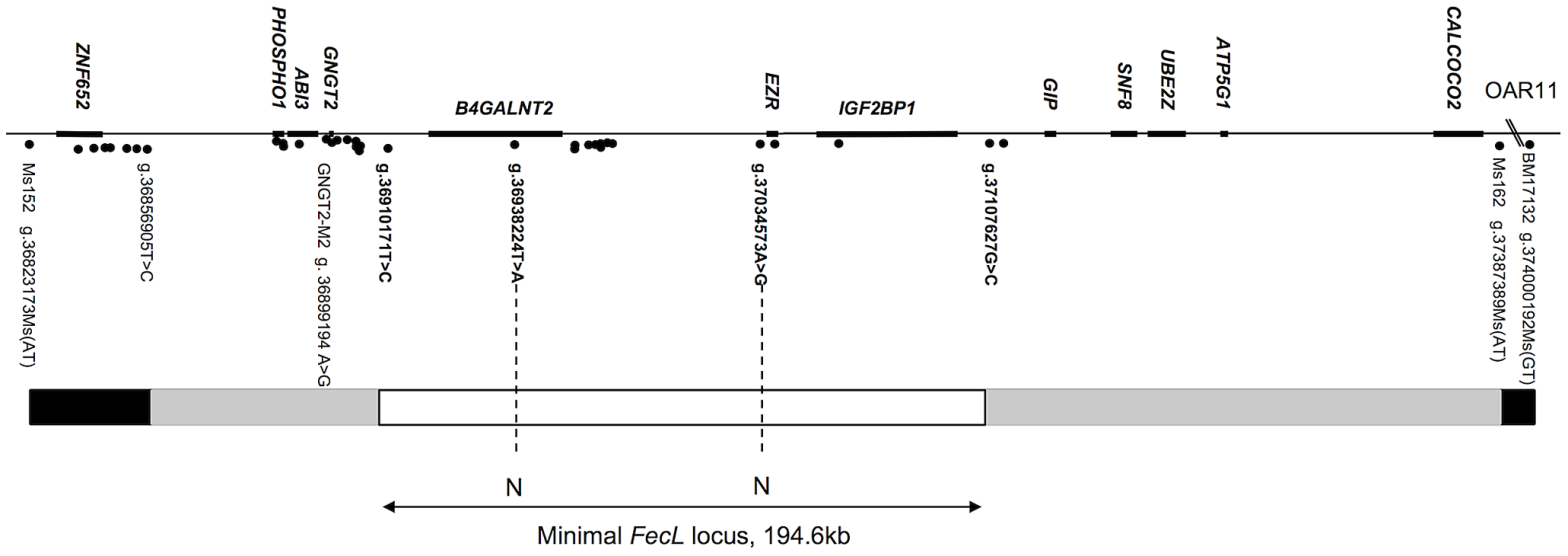
Map of the *FecL* locus on ovine chromosome 11. The genes are indicated above the line, markers are indicated by points under the line. The *FecL* locus (197 kb on OARv3.1, or 194.6 kb, our own sequencing) is flanked by the two closest recombinant markers, g.36910171T>C and g.37107627G>C. Recombinants: white box, zero-recombinant zone; gray boxes, zone with one recombinant; black boxes, at least two recombinants with *FecL*. N: no Allele sharing between wild-type and carrier animals for the *FecL^L^* allele.

The interval of localization previously published corresponded to a synteny block of 1.1 megabases on HSA17 [8]. Genotyping additional markers further reduced it across the whole experimental Lacaune pedigree (F1, BC and F1xBC representing 189 animals). This reduced interval was comprised between markers GNGT2-M2 (OAR11:36899194) and Ms162 (OAR11:37387389) encompassing 488 kb on the ovine chromosome 11 (OAR11, ovine genome version 3.1 released October 2012) and corresponded to a block of synteny of 479 kb on bovine chromosome 19 (BTA19, bovine genome version 4.6.1 released October 2011). This entire region was sequenced with the Roche 454 sequencing technology, using long-range PCR, in one heterozygous *L/+* animal and two homozygous *+/+* and *L/L* animals. Sixty-two polymorphisms were evidenced and an appropriate subset was genotyped on the recombinant animals allowing the reduction of the locus. This new interval of localization, comprised between two SNP markers on OAR11 g.36910171T>C (recombinant ewe n°990855) and g.37107627G>C (recombinant ewe n°60718), was estimated at 197 kb based on ovine genome OARv3.1 (Figure 1). This region encompasses 3 predicted protein-coding genes on the ovine genome, named *B4GALNT2* (beta-1,4-N-acetyl-galactosaminyl transferase 2), *EZR* (ezrin) and *IGF2BP1* (insulin-like growth factor 2 mRNA binding protein 1).

### Screening of the polymorphisms fully associated with the FecL^L^ mutation

In order to identify all the polymorphisms contained within this 197 kb interval, we analyzed separately the sequences of the two homozygous *L/L* and *+/+* animals. Sequence information coming from the Roche 454 sequencing technology was contained within 13 and 17 independent sequence contigs in *L/L* and *+/+* animals, respectively. We have then completed these sequences by Sanger sequencing to link contigs with each other. By comparing the two sequences, we identified 49 polymorphisms (43 SNP, 4 microsatellites and 2 Insertion/Deletion, Table 1). None of the detected variants affected the coding sequence of the annotated genes. Polymorphisms were first tested on a set of one *L/L*, one *L/+* and 2 to 6 *+/+* animals for allele sharing. If the allele associated with the L-haplotype was not found on a wild chromosome, then the marker was tested on successive subsets of wild chromosomes from the Lacaune families and other sheep populations, as described in the materials and methods section. There were only two polymorphisms segregating as the *FecL^L^* mutation, i.e. fully associated with the hyperprolific phenotype: the SNP g.36938224T>A, localized in the intron 7 of *B4GALNT2*, and the SNP g.37034573A>G localized in the intergenic sequence between *B4GALNT2* and *EZR*, 10.4 kb upstream of *EZR* (Figure 1).

**Table 1:** Polymorphisms within the minimal *FecL* locus and allele sharing.

OAR11 position	*FecL* locus position	Polymorphism type (+>L)	Allele sharing	OAR11 position	*FecL* Locus position	Polymorphism type	Allele sharing
36910171	1	SNP T>C	0/93^a^	36978101	66090	SNP A>G	10/13
36910432	262	SNP T>C	1/5	36978780	66769	SNP T>C	10/13
36910438	268	SNP C>T	1/5	36979857	67846	SNP C>T	6/13
36910484	314	SNP G>A	1/5	36980000	67989	SNP G>T	6/13
36910629	459	SNP C>T	1/43	36980361	68350	SNP T>C	3/9
36911198	1038	SNP C>T	5/23	36980408	68397	SNP A>G	5/9
36912873	2713	SNP C>T	3/19	36980459	68448	SNP A>C	6/13
36912938	2778	2778_2779del TT	11/19	36980802	68790	SNP G>A	6/13
36913274	3114	SNP T>A	4/19	36983172	71223	SNP T>G	6/13
36913693	3533	SNP T>C	4/167	36983502	71553	SNP G>A	6/13
36913712	3551	SNP A>G	4/167	36983554	71605	SNP G>C	8/13
36913851	3690	SNP A>G	4/125	36983772	71823	SNP T>C	8/13
36914601	4440	SNP G>A	1/71	36983838	71889	SNP A>G	8/13
36914685	4523	SNP T>G	7/71	37002721	90747	Ms ACC(10_?)	11/13
						AAC(11_?)^c^	
36914775	4613	SNP C>T	6/75	37009592	97825	Ms TG(19_20)	60/80
36914951	4789	SNP G>A	1/19	37013699	102307	SNP G>A	1/19
36915281	5118	SNP A>G	4/19	37022949	111585	SNP T>C	16/19
36934758	24609	SNP T>C	17/19	**37034573**	**123335**	**SNP A>G**	**0/602** b
**36938224**	**28075**	**SNP T>A**	**0/602** b	NA	135142	SNP G>A	19/24
36954793	43665	SNP T>G	1/40	NA	135152	135152_13529 delinsTGTACGAGT	10/22
36970424	58415	Ms GT(13_?)^c^	11/13	37064535	151388	SNP G>T	17/19
36973492	61488	SNP G>A	9/15	37065546	152517	SNP T>C	9/13
36973984	61978	SNP T>C	9/13	37095901	182887	Ms AC(12_?)^c^	3/13
36974191	62185	SNP T>A	7/13	37107627	194639	SNP G>C	1/19^a^
36974440	62434	SNP C>T	25/30				

Polymorphism positions are given relative to OAR11 v3.1 and *FecL* locus (Genbank:KC352617). Polymorphism type, single nucleotide polymorphism (SNP), microsatellite (Ms) or insertion/deletion (ins/del) referred to *+/+* vs. *L/L* sequence. Allele sharing indicated number of (*+*) chromosomes carrying the (*L*) allele relative to the number of (*+*) chromosomes tested.

a SNP corresponding to the interval borders and therefore excluded from the minimal locus.

b Allele segregating as the *FecL^L^* mutation.

c Ms marker genotyped by fluorescent SSCP, no information on the dinucleotide repeat variation.

NA, non-available coordinate on OARv3.1.

The sequencing of the locus indicated a real interval of 194.6 kb (GenBank:KC352617) and allowed to correct and complete the ovine reference genome sequence in this region. The locus effectively contained the full sequence of the *B4GALNT2* gene based on the ovine *B4GALNT2* mRNA (sequenced from Lacaune sheep, GenBank:KC175557), the full sequence of the *IGF2BP1* gene based on the bovine *IGF2BP1* mRNA (GenBank:NM_001192454) and a BLAST hit with the bovine *EZR* mRNA (GenBank:NM_174217) that we assumed to be a pseudogene. Indeed, the bovine *EZR* gene is located in a region of BTA9 that was not syntenic of the ovine *FecL* locus on OAR11 and the predicted sequence of this *EZR* annotation on OAR11 carried a premature STOP codon limiting the predicted protein to 223 amino-acids instead of 581. Consequently, only *B4GALNT2* and *IGF2BP1* were considered as positional candidate genes for *FecL*.

### Gene expression analysis

**Figure 2:**
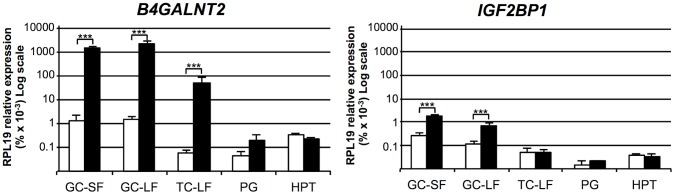
Expression of genes within the minimal FecL locus. Total RNA from granulosa cells (GC) from small (SF, 1–3 mm), granulosa cells and theca cells (TC) from large (LF, ≥6 mm) follicles, pituitary gland (PG) and hypothalamus (HPT) were reverse-transcribed and submitted to real-time PCR analysis for quantification of *B4GALNT2* and *IGF2BP1* gene expression. Data are means ± SEM of relative expression to the reference gene *RPL19* showed on a log scale. Asterisk indicates significant difference between means (n = 5) from non-carriers (*+/+*) and homozygous carriers of the *FecL^L^* mutation (*L/L*), **: p<0.01; ***: p<0.001.

The mRNA expression of the 2 positional candidate genes within the *FecL* locus was checked by real-time quantitative PCR in different tissues of the reproductive axis (hypothalamus, pituitary gland and ovarian granulosa and theca cells) isolated from *+/+* and *L/L* ewes. In the hypothalamus and the pituitary gland, *B4GALNT2* and *IGF2BP1* mRNA amounts were similar between genotypes, but they were significantly higher in the ovarian cells of the *L/L*, compared to +/+ ewes (Figure 2). Interestingly, *B4GALNT2* exhibited a 1000-fold higher expression level in the *L/L* granulosa and theca cells, whereas *IGF2BP1* expression was enhanced 6-fold only in granulosa cells and was found unchanged in theca cells. To check if this over-expression might concern other genes of the locus, the expression of the flanking genes within the “one recombinant zone” (Figure 1), i.e. *PHOSPHO1*, *ABI3* and *GNGT2* on the one side and *GIP*, *SNF8*, *UBE2Z*, *ATP5G1 and CALCOCO2* on the other side, was also studied. None of these genes showed an altered expression in the different *L/L* tissues of the reproductive axis. Given that *GIP* was not expressed in those tissues, its expression was checked in intestine and no difference was found between genotypes (supplemental figure S1). Moreover, as IGF2BP1 is a RNA binding protein controlling the steady-state level of the *MYC* oncogene mRNA [11] and the *ß-actin* (*ACTB*) gene translation [12] we checked for *MYC* mRNA expression and ACTB protein accumulation in Lacaune granulosa cells as a possible consequence of IGF2BP1 overexpression (supplemental figure S2). Real-time PCR analysis showed a large decreased expression of the *MYC* gene in large follicles compared to small ones (18-fold, p<0.001), but no alteration associated with elevated *IGF2BP1* mRNA level in *L/L* granulosa cells. ACTB accumulation checked by western blotting was not affected by the *L/L* genotype either.

Thus, the localization of the *B4GALNT2* gene within the minimal locus, the presence of the SNP g.36938224T>A on OAR11, localized in the intron 7 of this gene as the possible causal mutation, and the presence of a very high overexpression of the gene in the ovarian cells of *L/L* ewes led us to further investigate B4GALNT2 ectopic expression and activity in the ovary of the *FecL^L^* carrier animals.

### Localization of B4GALNT2 protein and its activity in the FecL^L^ ovary

**Figure 3:**
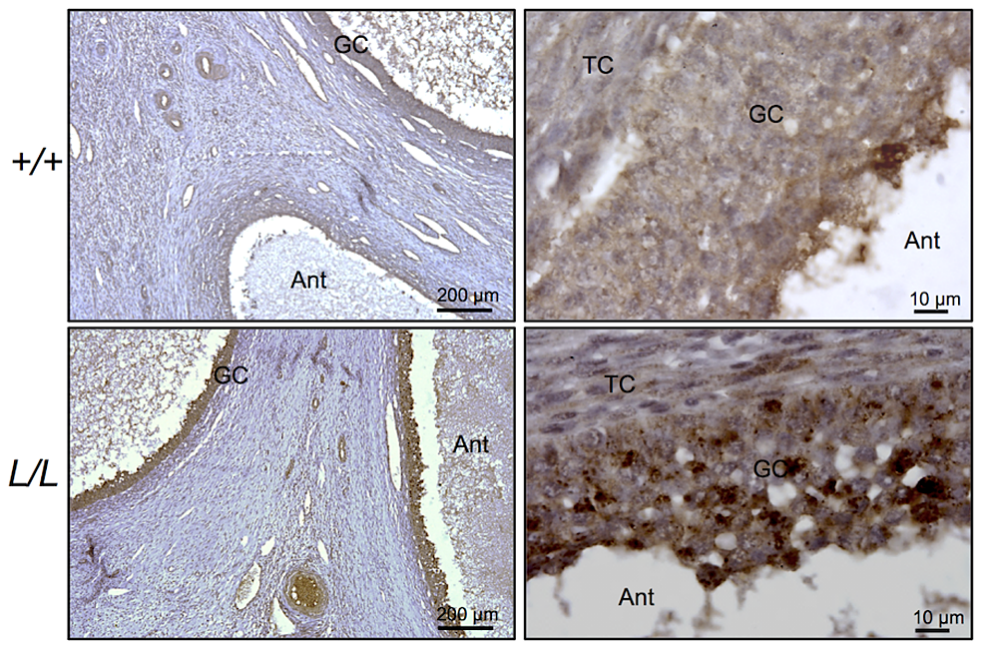
Immunostaining for B4GALNT2 in Lacaune sheep ovary. Photomicrographs of ovarian sections from +/+ and L/L ewes stained with anti-B4GALNT2 rabbit polyclonal antibody (1/50 dilution). Sections were counterstained with hematoxylin. A black segment indicates the microscopy magnification scale. GC, granulosa cell layer; TC, theca cell layer; Ant, antral cavity.

**Figure 4:**
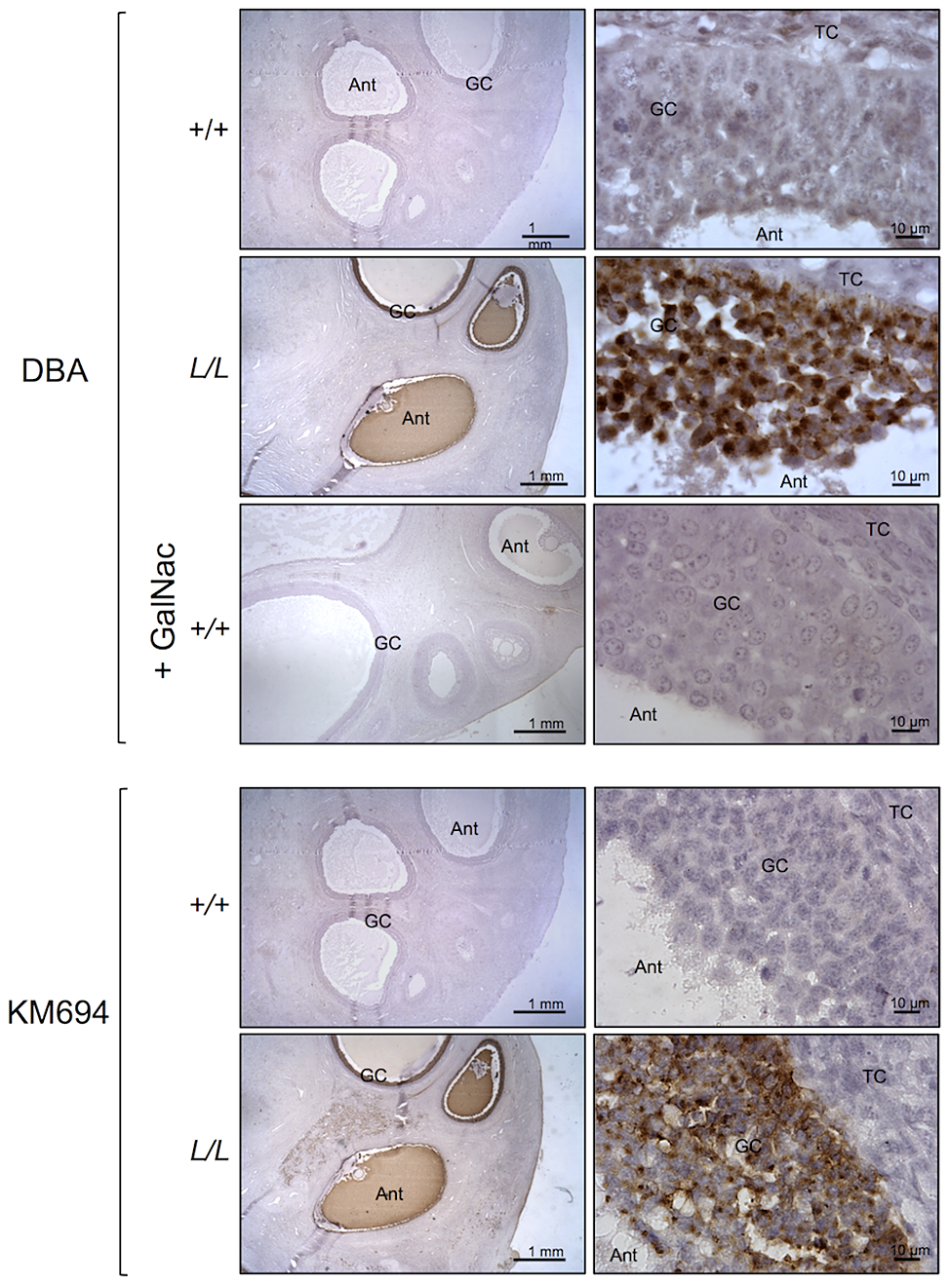
B4GALNT2 transferase activity revealed by DBA lectin and KM694 antibody staining in Lacaune sheep ovary. Photomicrographs of ovarian sections from +/+ and L/L ewes and stained either with biotinylated-DBA lectin (500 ng/ml) or KM694 mouse monoclonal antibody (1/1000 dilution). A GalNac treatment (200 µM) was used to compete for DBA staining as specificity control. Sections were counterstained with hematoxylin. A black segment indicates the microscopy magnification scale. GC, granulosa cell layer; TC, theca cell layer; Ant, antral cavity.

**Figure 5:**
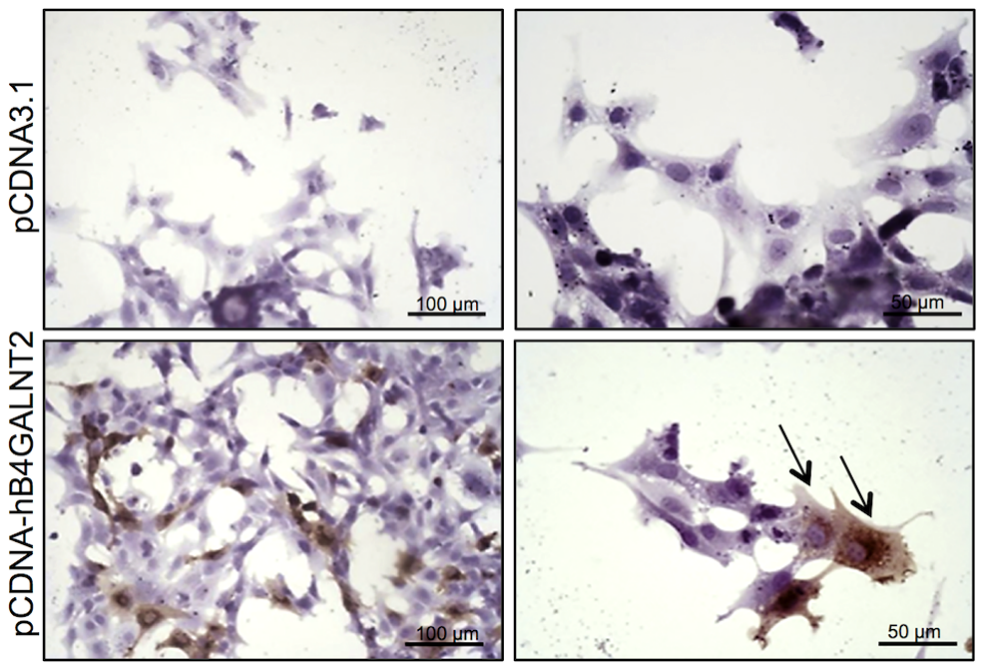
B4GALNT2 transferase activity revealed by DBA lectin after in vitro overexpression of *B4GALNT2* in ovine granulosa cells. Primary ovine granulosa cells from *+/+* small antral follicles were transiently transfected with either the pCDNA-hB4GALNT2 expressing the human form of B4GALNT2 or the empty pCDNA3.1 vector. Twenty-four hours after transfection, cells were stained with biotinylated-DBA lectin (500 ng/ml). Arrows indicated DBA positive staining only in B4GALNT2 transfected cells. Cells were counterstained with hematoxylin. A black bar indicates the microscopy magnification scale.

B4GALNT2 is a β1,4-N-acetylgalactosaminyltransferase which was previously shown to be involved in the synthesis of the Sd(a) antigen, a carbohydrate expressed on erythrocytes, colonic mucosa and other tissues. B4GALNT2 transfers a beta-1,4-linked GalNAc to the galactose residue of an alpha-2,3-sialylated chain found on both N- and O-linked glycans [13]. Immunohistochemistry experiments using an antibody raised against B4GALNT2 showed that the enzyme was detectable in the granulosa cells of *L/L* ovarian follicles only (Figure 3). The use of the *Dolichos Biflorus* Agglutinin (DBA) lectin and the KM694 antibody raised against the Sd(a) antigen, both specific of the glycosylation activity of B4GALNT2, clearly localized the targets of the enzyme only in the granulosa cells and the antral follicular fluid of *L/L* ovaries (Figure 4). To confirm the glycosylation activity of B4GALNT2, *+/+* ovine granulosa cells were transiently transfected by a *B4GALNT2* expressing construct. Specific staining with DBA was observed in *B4GALNT2*-transfected *+/+* granulosa cells (Figure 5), indicating that overexpression of the *B4GALNT2* gene is directly related to positive DBA staining.

**Figure 6:**
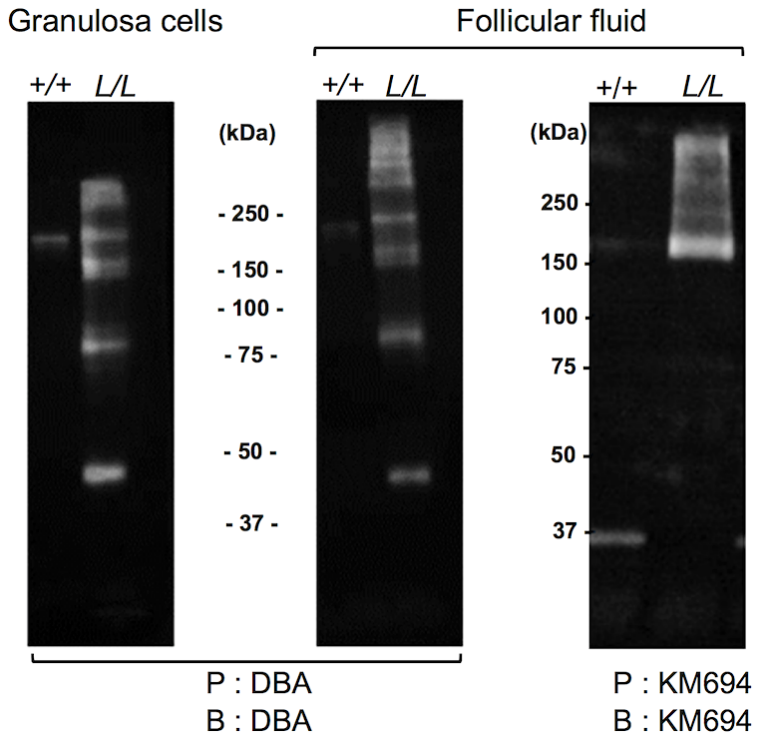
Western immunoblotting analysis of B4GALNT2 transferase activity in Lacaune sheep granulosa cells and follicular fluids. Granulosa cell protein extracts (50 µg) and follicular fluids (200 µg) from *+/+* and *L/L* large antral follicles were precipitated (P) by agarose-DBA lectin or sepharose-protein A-KM694 monoclonal antibody. The resulting purified glycoproteins were separated on SDS-PAGE, transferred on nitrocellulose membrane and revealed after blotting (B) using biotinylated-DBA lectin or KM694 monoclonal antibody.

The presence of a B4GALNT2 activity in the granulosa cells of the *FecL^L^* carrier ewes was confirmed by the results of DBA lectin and KM694 antibody precipitation and subsequent western-blot experiments using *L/L* and *+/+* granulosa cell extracts and follicular fluids (Figure 6). Indeed, DBA or KM694 staining revealed very different glycoprotein profiles between *L/L* and *+/+* ewes. In both granulosa cells and follicular fluids, at least 7 glycoprotein forms of various molecular weights (ranging from 40 to over 250 kDa) were retained by DBA lectin precipitation in the *L/L* ewes and were absent in non-carrier ewes. The KM694 immunoprecipitation of the proteins contained in the follicular fluids of the *FecL^L^* carrier ewes evidenced a subset of these glycoproteins, with a high molecular weight.

### Mass spectrometry identification of the glycosylated targets of B4GALNT2

We aimed at identifying the glycoproteins which are the targets of B4GALNT2 in ovary after their purification from *L/L* follicular fluids using DBA lectin affinity, followed by high-resolution mass spectrometry. Peptides and proteins purified from *L/L* and *+/+* follicular fluids were identified with at least four independent peptides and a high probability threshold (>95%). This allowed identifying, on two repetitions of the experiment, 10 glycoproteins only present in *FecL^L^* follicular fluids, and then suspected to be the main substrates of B4GALNT2 activity (Table 2). Identified proteins were of a large molecular weight range (ranging from 39 to 613 kDa) and most of them were extracellular matrix or membrane-associated proteins. Interestingly, the inhibin α and βA subunits, leading to the production of Activin A and Inhibin A, and the chondroitin sulfate proteoglycan Versican are well known to be directly involved in female reproductive function [14]–[16]. These proteins represent promising physiological candidates to understand the mechanism by which the *FecL^L^* mutation affecting *B4GALNT2* expression increases the ovulation rate in Lacaune sheep population.

**Table 2:** DBA-purified proteins present in L/L follicular fluid identified by mass spectrometry.

Identified proteins	HUGO gene symbol	UniProt Acc.Number	Theoretical Molecular Weight (kDa)
Hemicentin-1	HMCN1	Q96RW7	613
Prolow-density lipoprotein receptor-related protein 1	LRP1	Q07954	505
Versican	VCAN	P81282	370
Coagulation factor V	F5	Q28107	249
Inter-alpha-trypsin inhibitor heavy chain H1	ITIH1	Q0VCM5	101
Heparan-sulfate 6-O-sulfotransferase 2	H6ST2	Q96MM7	69
Clusterin	CLU	P17697	51
Inhibin, beta A	INHBA	P07995	48
Glia-derived nexin (serpin peptidase inhibitor, clade E, member 2)	SERPINE2	P07093	44
Inhibin, alpha	INHA	P07994	39

## Discussion

### Genetic characterization of the FecL locus

The fine mapping strategy combining marker development through high-throughput sequencing and genotyping of selected recombinant animals allowed the delimitation of the *FecL* locus on OAR11 between SNP markers g.36910171T>C and g.37107627G>C. With the double aim to densify the locus with more informative polymorphisms and to directly identify the causal polymorphism, we developed a systematic high-throughput sequencing of the locus with the Roche 454 technology on finely chosen animals. The targeting of the locus was done by overlapping long-range PCR fragments (around 10 kb), a method successfully used to detect genomic variations in *BRCA1/BRCA2* locus in human [17]. We experienced some bias in detecting polymorphisms, especially SNP, in the reads depth of the L/+ animal due non-independent reads [18] and PCR-dependent allele disequilibrium. Nevertheless the use of the two independent sequences *L/L* and *+/+* allowed the elimination of this latter bias. The polymorphisms evidenced were further studied for allele sharing. The different appropriate subsets of markers that were genotyped on recombinant ewes led to the reduction of the minimal interval to 194.6 kb. Within this interval of localization, 2 SNPs have been found fully associated with the *FecL^L^* mutation, namely SNP g.36938224T>A and SNP g.37034573A>G. The SNP g.36938224T>A was localized in the intron 7 of *B4GALNT2*, in a sequence portion that was only conserved in the bovine orthologous gene, and not in other species. The SNP g.37034573A>G was localized in the intergenic sequence between *B4GALNT2* and *IGFBP1* in a non-conserved region enriched with LINES and SINES repetitive elements.

The finding of non-coding SNPs as causal mutations contrasts with other known mutations affecting ovulation rate through alteration of the coding sequence of *BMP15*, *GDF9* and *BMPR1B* genes impairing the protein function [10]. To go further in the fine mapping study, different strategies can be proposed. The first one is to find additional recombinant animals within the genetic families. A second one is to find recombinant animals at the population level, i.e. animals carriers of a shorter L-haplotype and finely phenotyped. However the probability to find such animal recombining within an interval of 195 kb is very low. If only one of these two SNP is the causal mutation, one way to prove it is to find an animal carrying a recombination between the two markers. However, the 2 SNPs are 96 kb apart and the probability to find such animal is nearly null. A third possibility is to increase the number of wild chromosomes (i.e. wild haplotypes) tested for allele sharing. Animals coming from 9 different breeds have been genotyped (representing 180 wild haplotypes) but did not allow the elimination of one or the other putative causal mutation.

### Expressional candidates of the FecL gene and effect of the SNPs

Annotation available on the last release of the sheep genome assembly (OARv3.1) indicated that the minimal 194.6 kb interval, that we entirely sequenced, contained only 3 potential protein encoding genes, *B4GALNT2*, *EZR* and *IGF2BP1*. The EZR annotation is very recent (October 2012) and this gene was not considered as an expressional candidate for *FecL* at the beginning of the work. Moreover, if the localizations of *B4GALNT2* and *IGF2BP1* on OAR11 were coherent with their syntenic location on BTA19 and HSA17, the presence of the *EZR* gene annotation is intriguing. Indeed, in bovine (UMD3.1) and human (GRCh37) genomes, this gene is positioned on BTA9 and HSA6, respectively (www.Ensembl.org). Moreover, Blastn analysis (www.livestockgenomics.csiro.au) of the bovine *EZR* cDNA (NM_174217) indicated sequences producing significant alignments on Ovis aries breed Texel contig_127582, OAR11 and OAR8. Only matched sequences on OAR8 presented an exon/intron structure in a syntenic region of BTA9 and HSA6, and then should correspond to the ovine *EZR* gene. The high identity match we found between bovine *EZR* cDNA and both the ovine reference genome on OAR11 and our own *FecL* locus sequence (98% coverage, 95% identity) evidenced the presence of an *EZR* spliced pseudogene with a premature stop codon impairing the translation of an EZR protein. For those reasons, only *IGF2BP1* and *B4GALNT2* were considered as positional candidate genes for *FecL* in this study.

Due to the absence of polymorphism in the coding sequence of *IGF2BP1* and *B4GALNT2* genes, we searched for an expressional candidate to discriminate between the two genes. Real-time PCR analysis showed that both *IGF2BP1* and *B4GALNT2* expression was significantly affected by *FecL^L^*, specifically in ovarian cells indicating a tissue-specific regulation by the *FecL^L^* mutation. Interestingly, no differential expression was observed for genes outside of the minimal locus, reinforcing the genetic fine mapping result. For follicles collected at the same size class, the differential expression observed between +/+ and *L/L* follicles could not be attributed to the existence of a difference in follicle maturity as already observed for the hyperprolificacy-associated Booroola mutation [19]. Indeed, *IGF2BP1* and *B4GALNT2* expression in granulosa cells was not different between small and large antral follicles that were at different maturity stages as attested by marker expression, such as *LHR*, *INHA*, *CYP19A1* and *CYP11A1* as previously shown [9] or *MYC* (present study).

SNP g.36938224T>A, localized in the intron 7 of *B4GALNT2*, and SNP g. 37034573A>G in the inter-genic region, were the only two polymorphisms segregating as the *FecL^L^* mutation. They were then thought to be directly the cause of the huge overexpression of *B4GALNT2* and in a lesser extent of *IGF2BP1* in the ovaries of *FecL^L^* carrier ewes, through a molecular mechanism that remains to be determined. In order to explain this differential expression we searched *in silico* for transcriptional factors able to bind these SNP locations. However, we failed to find matches with consensus sequences binding known transcriptional factors at each site. Experimentally, electromobility shift assays with ovine granulosa cell nuclear extracts failed also to discriminate between SNPs and between prolific and wild type alleles. As a second *in silico* approach, we also searched matches in Patrocles, the database of polymorphic microRNA-target interactions (www.patrocles.org). Polymorphism in miRNA target sites might be important effectors of phenotypic variation as attested by the hypermuscularity phenotype in Texel sheep [20]. Interestingly, the *B4GALNT2* intronic SNP g.36938224T>A was found to create a motif GTGTGAGA in *FecL^L^* which is a recognition site for MIR342 that is conserved among bovine and human. However, we cannot reconcile this finding with the current knowledge on gene regulation by microRNAs, indicating that those small noncoding RNAs usually targeted matured mRNA in the 3′-untranslated regions within cytoplasmic protein complexes to repress protein synthesis [21]. Another hypothesis is that the intronic location of the SNP g.36938224T>A may be associated with alternative splicing of the *B4GALNT2* mRNA, but analysis by RT-PCR amplification between exon 6 and terminal exon 11 failed to evidence such alternative splicing between *L/L* and *+/+* mRNA. In human gastrointestinal cancer cells the expression of *B4GALNT2* is dependent on the promoter methylation status [22]. As in other species, a large CpG island is present in the vicinity of the ovine *B4GALNT2* promoter. By bisulfite sequencing or restriction site analysis by methyl sensitive enzymes of granulosa cell derived DNA from +/+ and L/L animals, we failed to detect any differential methylation status. Thus, it remains to be determined how the g.36938224T>A and/or g.37034573A>G SNPs are able to distantly regulate *B4GALNT2* and *IGF2BP1* expression at the mRNA level specifically in ovarian cells, maybe through yet unknown tissue-specific DNA-protein interactions.

### Role of the IGF2BP1 gene in the ovarian function

The *IGF2BP1* gene was part of the minimal *FecL* locus and its expression was increased under the influence of the *FecL^L^* prolific allele (being g.36938224A and/or g.37034573G) in granulosa cells. This expression in ovine ovary is consistent with the *IGF2BP1* expression already observed in mouse and human ovaries [23]. Elevated *IGF2BP1* expression was also associated with ovarian carcinoma and proliferation deregulation through a c-myc (MYC) dependent mechanism [24]. Indeed, IGF2BP1 possesses RNA binding motifs and can stabilize the *MYC* mRNA [11] and enhance the translation of several other genes such as *IGF2* and *ß-actin* (*ACTB*) by binding to their mRNA [25]. Interestingly, all these known target genes of IGF2BP1 action play an important role in follicle function. IGF2 is present in follicular fluid [26], expressed by granulosa [27] and theca cells [28] and participates in the maturation of ovarian follicles [29]. Actin is associated with granulosa cell shape changes along antral folliculogenesis [30] that may consequently affect steroid synthesis and proliferation [31]. MYC participates in the control of granulosa cell proliferation under gonadotropin and insulin stimulation [32], [33]. However, checked by *MYC* mRNA and ACTB protein accumulation, it seemed that the 6-fold overexpression of *IGF2BP1* had no biological consequences in the granulosa cells of *L/L* ovaries.

### Role of the B4GALNT2 gene in the increased ovulation rate of the FecL^L^ ewes

The *FecL^L^* prolific allele was clearly associated with an ectopic overexpression (at least 1000-fold compared to wild-type) of *B4GALNT2* mRNA in ovarian granulosa and theca cells from antral follicles. This ectopic expression seemed to occur specifically in the ovary, since expression checked by real-time qPCR in pituitary, hypothalamus (Figure 2), intestine (Figure S1) and adrenals (data not shown) showed no difference between genotypes, indicating a tissue-specific mechanism for *B4GALNT2* expression regulation by the *FecL^L^* mutation. Such ectopic expression of *B4GALNT2* has never been demonstrated in the ovary before. However, ectopic expression of *B4galnt2* is the molecular basis for the action of the Mvwf locus, a major modifier of plasma von Willebrand factor (VWF) level in RIIIS/J mice [34]. Indeed a switch of *B4galnt2* gene expression from intestine epithelial cells to vascular endothelial cells, resulting in aberrant VWF glysosylation, explained the phenotypic characteristics of the RIIIS/J mice similar to human type 1 von Willebrand disease. The region responsible for the Mvwf locus regulatory switch lies within a 30-kb genomic interval upstream to the *B4galnt2* gene [35]. In the present study, one of the potential causal SNP (g.37034573A>G) lies 42 kb upstream to *B4GALNT2* in a region which is not conserved between sheep and mouse.

The mRNA huge overexpression of *B4GALNT2* observed in the *L/L* ovaries was accompanied by increased protein expression level as shown by specific immunohistochemistry, mainly in granulosa cells. As previously stated, B4GALNT2 is involved in the synthesis of the Sd(a) antigen on various protein targets [13] with the transfer of a terminal GalNAc, specifically recognized by DBA lectin [34] and the KM694 antibody [36], [37]. Using DBA lectin staining, we evidenced the GalNac transferase activity exclusively in the granulosa cells of the *FecL^L^* ovaries. The glycosylated targets of B4GALNT2 were mainly secreted in the follicular fluid as shown by histochemistry and western-blotting experiments. However, the pattern of target proteins revealed by DBA lectin and KM694 antibody was different. This discrepancy could be explained by the strict recognition specificity of the Sd(a) antigen (GalNac transfer to terminal α2,3-sialylated galactose residue in the ß1,4 linkage) by the KM694 antibody but a wider spectrum of terminal GalNac recognition by DBA lectin, indicating that B4GALNT2 could create other carbohydrate structures than the Sd(a) antigen. Moreover, we demonstrated that the *in vitro* overexpression of B4GALNT2 was responsible for the DBA lectin staining. Regarding these results, we assume that an atypical glycosylation of proteins within the granulosa cells of *FecL^L^* ewes, which does not occur in wild-type cells, is due to the overexpression of B4GALNT2. This could represent the initiating mechanism of the increased ovulation rate which characterizes the *FecL^L^* Lacaune sheep. In transgenic mice, manipulating glycosylation at the oocyte level led to slight increased fecundity or primary ovarian insufficiency depending on the glycosyltransferase being invalidated [38], [39]. Anyway, it proves the importance of glycosylation in the control of ovarian function.

In order to go further in the role of B4GALNT2 in the *FecL^L^* ovaries, we tried to identify the target proteins recognized by the DBA lectin using lectin affinity purification. Through comparative mass spectrometry analysis, we identified several atypically glycosylated proteins secreted in the follicular fluid of *L/L* ewes. Among the glycoproteins identified, were the inhibin subunits (INHA and INHBA) participating in the inhibin A and the activin A hormone formation [16]. Inhibin A and activin A are dimeric glycoproteins belonging to the transforming growth factor-beta (TGFβ) superfamily. They are produced by granulosa cells and can accumulate in high concentrations in follicular fluid. Activin A is considered to act mainly through auto/paracrine signaling in granulosa and theca cells [16], while inhibin A acts mainly through endocrine negative feedback regulation of pituitary FSH secretion. However, inhibin A has also been shown to exert a blocking action on the activin A and other TGFß member dependent regulation of steroidogenesis and proliferation within the ovary [16]. Immunization against inhibin can promote an increase in ovulation rate and prolificacy [40] through enhanced follicular development in sheep [41], goat [42] and water buffalo [43]. It has been suggested that inhibin antibodies may act primarily by an intraovarian paracrine action rather than by reducing the suppressive action of inhibin on pituitary FSH release [43], [44]. Inhibin A is a heterodimer of α- (INHA) and ßA- (INHBA) subunits, whereas activin A is a homodimer of ßA subunits. Interestingly, the differential subunit association (α-ßA or ßA-ßA) was dependent on glycosylation events implicating N-linked oligosaccharide [45]. The B4GALNT2 atypical glycosylation of INHA and INHBA could alter the subunits association, and then change the biological activity or the physiological ratio of activin A and inhibin A produced by the *FecL^L^* ovaries. Given that no difference in FSH plasmatic concentration during the follicular phase was observed between *+/+* and *L/L* Lacaune ewes [9], one might suspect a more direct consequence on activin A signaling within the ovary. Experiments are ongoing to demonstrate the direct effect of B4GALNT2-dependent glycosylation on inhibin A and activin A biological activity at the auto/paracrine and endocrine levels.

Other good physiological candidates to explain increased OR in Lacaune sheep were the proteoglycans versican (VCAN) and inter-alpha trypsin inhibitor (heavy chain H1, ITIH1), implicated in follicular fluid formation and osmotic gradient, cumulus expansion, follicular remodeling and finally fertility [15], [46]. The coagulation factor F5, the serine protease inhibitor of the serpin family SERPINE2 and the heparan sulfate proteoglycan sulfotransferase (HS6ST2) would be other important regulators of coagulation, controlling antithrombin, plaminogen or fibrinogen activities present in follicular fluid [47]–[51]. Clusterin (CLU), a sulfated glycoprotein can also act on the ovarian function through its protective effect on granulosa cells against apoptosis during follicular atresia [52], and it is well known that a reduction in atresia is associated with increased ovulation rate [53]. Clusterin is a binding protein of the low-density lipoprotein receptor-related protein-2 (LRP2) [54], and may participate in cholesterol delivery to steroidogenic cells as LRP8 does in the bovine ovary [55]. Interestingly, in Lacaune sheep follicular fluids, we evidenced the presence of LRP1 that could have the same function on cholesterol uptake but can also act as a TGFß receptor (type 5) and regulate its signaling in ovarian cells [56]. Hemicentins are extracellular matrix proteins implicated in cell contacts, adhesion and migration [57]. Hemicentin-1 (HMCN1) carries a von Willebrand A domain that may explain its glycosylation by B4GALNT2. Hemicentins interact with fibulins that are estrogen regulated and overexpressed in ovarian cancer cells [58], [59], but no direct role of hemicentins has been described in the ovarian function. Finally, all the glycoproteins identified could have a role in the ovarian follicle function, may be through the control of intra-follicular activity of hormones or growth factors and/or their transfer outside of the follicle to the general blood circulation.

In conclusion, the present study reports strong evidence for the *B4GALNT2* gene to be the *FecL* fecundity gene in Lacaune sheep. We propose that its overexpression in granulosa cells under the influence of only 1 or 2 non-coding regulatory SNP can induce an atypical glycosylation of follicular target proteins such as inhibin subunits and that is the starting point of the mechanism explaining increased ovulation rate and prolificacy in this breed. For the first time a fecundity gene in sheep does not belong to the TGFß/BMP signaling genes and it opens new fields of investigation regarding ovarian glycosylation and the pathogenesis of fertility disorders in women.

## Materials and Methods

### Animals

The presence of the Lacaune autosomal fecundity locus and its prolific allele *FecL^L^* was checked in our experimental Lacaune meat strain flock (n = 189) as described [8]. The three genotypes at the *FecL* locus are called *+/+*, *L/+* and *L/L* representing *FecL^+^/FecL^+^, FecL^L^/FecL^+^ and FecL^L^/FecL^L^*, respectively. Briefly, as a unique haplotype is associated with the *FecL^L^* mutation, the presence of this particular mutant haplotype was established by the genotyping of four close markers, including the DLX3:c.*803A>G SNP that alone provides accurate classification of animals (99.5%) as carriers or non-carriers of the mutation. The absence of the *FecX^L^* mutation was checked in the studied Lacaune ewes by direct genotyping of the mutation [3]. The estrus cycles of all adult Lacaune ewes were synchronized with intravaginal sponges impregnated with fluorogestone acetate (FGA, 40 mg, Intervet, Angers, France) for 14 days. All procedures were approved by the “Direction Départementale des Services Vétérinaires de Haute-Garonne” (approval number C31-429-01) for the agricultural and scientific research agency INRA (French National Institute for Agricultural Research), and conducted in accordance with the Guide for the Care and Use of Agricultural Animals in Research and Teaching.

### Roche 454 sequencing and data mining

Genomic DNA was extracted from blood samples following a salt-based DNA extraction [60]. Firstly, 45 long-range PCR fragments spanning 488 kb were amplified on genomic DNA from the heterozygous L/+ ewe n°982140, chosen as a dam of a double-recombinant ewe able to reduce the locus and because of its high level of homozygosity within the *FecL* locus. On a second time, 25 long-range PCR fragments spanning 250 kb were amplified from a homozygous *L/L* ewe and a homozygous *+/+* ewe. The *L/L* ewe n°991012 was chosen at random, as it exists only one L-haplotype in the studied population. The +/+ ewe n°011182 was chosen for its homozygosity all along the locus because several wild-type haplotypes segregated in the population. Long-range PCR fragments were amplified on an ABI 9700 thermocycler (Applied Biosystems) using the Long PCR Enzyme Mix (Fermentas). Independently for each animal, the resulting fragments were purified and pooled all together at equal concentrations. These samples were then sequenced using the Roche 454 Life Sciences Genome Sequencer FLX (454 Life Science, Roche), following the manufacturer's instructions. Three shotgun libraries were prepared with 1 µg of pooled PCR product DNA using the Titanium General Library Preparation Kit. Nebulized, purified, and adaptor-linked DNA fragments were amplified using the GS FLX Titanium LV emPCR Kit, and sequencing on the FLX Genome Sequencer was performed using the GS FLX Sequencing Kit, Titanium Reagents XLR70. *L/+*, *L/L* and *+/+* sequencing data from fastq files generated by 454 sequencer were cleaned using an in-house algorithm. A total of 356 873 reads with an average length of 366 bases were aligned on the sheep genome (OAR v2.0 - released March 2011 - draft sheep reference genome) with bwa software (bwasw algorithm [61], indicating a mean sequencing deepness of 103X. The resulting SAM format files were processed using samtools view, sort and merge functions [62]. Screening of polymorphisms was done “manually” using IGV software [63]. The position of all described polymorphisms within this manuscript is given according the last release of the sheep genome (OAR v3.1 - released October 2012 - sheep reference genome [64]).

### Marker analysis, genotyping and Sanger sequencing

Prior to 454 sequencing, the search for single nucleotide polymorphisms (SNPs) was performed from ovine ESTs and BAC end sequence information, on a set of 2 L/L and 8 +/+ animals by single strand conformation polymorphism (SSCP) and silver staining as described [8]. Identified polymorphic fragments, after SSCP or 454 sequencing, were amplified by PCR on ABI 9700 thermocycler (Applied Biosystems). Microsatellites genotyping through fluorescent SSCP was performed on an ABI 3100 sequencer (Applied Biosystems). Depending on markers, SNP genotyping was done by SSCP and silver staining, or SSCP with fluorescent primers as described in Applied Biosystems Publication 116AP01-02, or by direct Sanger sequencing using the ABI Prism BigDye Terminator v3.1 Cycle Sequencing kit (Applied Biosystems) on ABI 3100 sequencer (Applied Biosystems). The particular *FecX^L^* mutation was genotyped by SSCP as described [3].

After identification, the allelic sharing of a given marker was tested first on a subset of at least 2 to 6 +/+ Lacaune ewes, then the marker was tested on successive subsets of wild chromosome providing from: the Lacaune genetic family (n = 103), the dairy Lacaune GEBRO population (n = 173) and the Blanche du Massif-Central (BMC) population (n = 148) that shared common ancestors. Additional wild chromosomes from 9 different breeds genetically unlinked to the Lacaune breed were also used (n = 180, Limousine, Bizet, Rava, Suffolk, Causse du Lot, Charmoise, Préalpes du Sud, Berrichon du Cher, Noire du Velay, 10 animals of each breed).

### Tissue collection

Estrus-synchronized ewes (+/+, n = 13 and L/L, n = 14) were slaughtered during the follicular phase 36 h after FGA sponge removal. Ovaries, pituitary gland and hypothalamus were collected from each animal. Pituitary gland and hypothalamus were immediately frozen in liquid nitrogen and stored at −80°C for further RNA extraction. Some ovaries were immediately placed in fixative Bouin's solution and embedded in paraffin for further histochemistry. Other ovaries were finely dissected to isolate individual antral follicles >1 mm in diameter. Once dissected, follicles were classified according to their size, small (1–3 mm) and large (≥6 mm) and with respect to genotype, independent of atresia. Granulosa cells and follicular fluids were recovered from small and large follicles as described previously [65], and theca layer was gently detached from the follicular wall of large follicles with forceps and washed in PBS to eliminate residual granulosa cells. Pools of each category of cells were established per animal and stored at −80°C for further RNA or protein extraction.

### RNA extraction, Reverse transcription and quantitative PCR

Total RNA from ovarian cells and from pituitary gland was isolated using Nucleospin RNA II or Nucleospin RNA L kit, respectively, according to the manufacturer's protocol (Macherey-Nagel). Total RNA from hypothalamus was isolated by urea/LiCl precipitation and phenol extraction [66]. All RNA samples were DNAse-treated to avoid genomic DNA contamination and diluted at 0.5 µg/µl in RNAse-free water. RNA (1 µg) was reverse-transcribed using Superscript II reverse transcriptase (Invitrogen). Real-time quantitative PCR was run on a LightCycler 480 system (Roche Diagnostic) using Power SYBR Green PCR Master Mix (Applied Biosystems) in 384 wells-plate as described [8]. The specific primer sequences (Table 3) used for each gene were designed using the Beacon Designer 7 software (Premier Biosoft International). For each primer pair, efficiency curves were generated using serial dilutions of cDNA in abscissa and the corresponding cycle threshold (Ct) in ordinate. The slope of the log-linear phase reflects the amplification efficiency (E) derived from the formula E = e^(−1/slope)^. Amplification efficiency obtained for each primer pair is indicated in Table 3. For quantification analysis, the Ct of target gene was compared with the internal reference gene *RPL19* encoding an ubiquitous ribosomal protein, according to the ratio R = [E_L19_^CtL19^/E_target_^Ct target^] expressed in percentage.

**Table 3:** Quantitative PCR primer sequences and their efficiency.

Gene	Primer sequences (5′→3′)	Efficiency (E)
*B4GALNT2*	AAGATTGAGGTGCTGGTGGATG	2.01
	TTAACGACGCCGCTGGTC	
*IGF2BP1*	CAAGAAGGGGCAGCACATTAAAC	1.98
	TGGAGTTTCAGGAGGAGCAATC	
*RPL19*	AATGCCAATGCCAACTC	1.95
	CCCTTTCGCTACCTATACC	

### Histochemistry

Paraffin embedded ovaries were serially sectioned at a thickness of 7 µm. For immuno-histochemistry, deparaffinized sections were subjected to antigen unmasking solution (Vector Laboratories) boiled for 3 min. After two 5-min washes with 0.1% saponin in PBS, sections were incubated at 4°C for 30 min with PBS containing 0.1% saponin and 0.3% H_2_O_2_ to remove endogenous peroxidase activity. After three 5-min washes in 0.1% saponin in PBS, sections were incubated at 4°C overnight with goat polyclonal anti-B4GALNT2 antibody (sc-107334, Santa Cruz Biotechnology) diluted 1∶50 or mouse monoclonal KM694 (anti-Sd^a^) antibody kindly provided by Shigeyuki Yamano (Kyowa Hakko Kirin Co., Japan) diluted 1∶1000 in PBS containing 0.1% saponin and 0.1% BSA. After three 5-min washes in PBS containing 0.1% saponin, sections were incubated for 2 hours at room temperature with the biotinylated secondary antibody (donkey anti-goat, Santa Cruz Biotechnology; donkey anti-mouse, Jackson ImmunoResearch Laboratories) diluted 1∶800 in PBS containing 0.1% saponin and 0.1% BSA, and washed thereafter. For lectin-histochemistry, deparaffinized sections were subjected to Carbo-Free blocking solution (Vector Laboratories) for 30 min. After a 5-min wash in PBS containing 0.05% Tween-20 (Sigma), sections were incubated with PBS containing 0.5 µg/mL of biotinylated DBA lectin (Dolichos Biflorus Agglutinin, Vector Laboratories) for 2 hours at room temperature, with or without 0.1 M of N-Acetyl-D-galactosamine (Sigma) to check for its specificity and then washed in PBS containing 0.05% Tween-20. For both approaches, stained sections were incubated with avidin-peroxidase conjugate from Vectastain Elite ABC kit (Vector Laboratories) and developed with 0.4 mg/ml DAB (3,3′-diaminobenzidine tetrahydrochloride dehydrate; Sigma) and 0.012% H_2_O_2_ in 50 mM Tris-HCl (pH 7.8) for 1 to 5 min at room temperature. Negative control sections involved omission of the primary antibody or the lectin from the procedure.

### Granulosa cell transient transfection

Granulosa cells from small antral +/+ follicles were seeded at 100 000 viable cells/chamber on Lab-Tek 8 chambers slide system (Thermo-Scientific) and cultured for 48 h at 37°C with 5% CO_2_, in McCoy's 5a medium (Sigma) supplemented with 3% fetal ovine serum (FOS). Cells were transiently transfected with 1 µg of empty pCDNA3.1 or pCDNA-hB4GALNT2 (kindly provided by A. Harduin-Lepers) using jetPEI transfection reagent (Polyplus Transfection) for 18 h with a DNA/JetPEI ratio of 1/2 (w/w) as specified by the manufacturer, and thereafter, medium was changed with fresh McCoy's 5a medium supplemented with 3% FOS for an extra 30 h. At the end of the culture period, cells were fixed in 4% paraformaldehyde for 10 min at 4°C. Slides were washed in PBS 0.1% saponin, and then stained with biotinylated DBA lectin (0.5 µg/µL) as described above.

### Lectin precipitation and western blotting

Granulosa cell whole cell extracts were obtained by resuspension in RIPA lysis buffer as previously described [67]. Granulosa cell lysates and follicular fluids from large antral follicles were centrifuged at 15000 g for 20 min at 4°C, and the protein concentration in the supernatant was determined by a colorimetric assay (BC Assay kit; Uptima Interchim). Protein samples corresponding to 50 µg of granulosa cell extract or 200 µg of follicular fluid were incubated with 30 µl of agarose-coupled DBA lectin (Vector Laboratories) in 300 µL of RIPA buffer for 2 hours at room temperature on a rotating platform. Alternatively, 250 µg of protein from follicular fluid were incubated with 25 µL of protein A-sepharose (Vector Laboratories) and KM694 antibody (1∶300). After a brief centrifugation (10000 g, 2 min), supernatant was discarded and the precipitated protein complex linked to agarose or sepharose beads was washed 3 times with 1 mL RIPA buffer. After wash, the complex was resuspended in Laemmli sample buffer (Bio-Rad), fractionated using SDS-PAGE in 10% polyacrylamide gels and transferred onto nitrocellulose membranes. Membranes were either blocked for 30 min at room temperature in Carbo-Free solution, and stained with 0.5 µg/mL biotinylated-DBA lectin (Vector laboratories) for 1 h at room temperature in PBS, or blocked with 5% non-fat dry milk, 0.1% Tween 20 (Sigma) in PBS, stained for 2 h with KM694 antibody (1∶1000), followed by incubation with peroxidase-conjugated anti-mouse IgG (1∶2500; Jackson ImmunoResearch Laboratories) for 1 h at room temperature. Glycoproteins precipitated by DBA lectin were revealed by ABC reaction (Vectastain, Vector Laboratories), followed by ECL Plus detection (GE Healthcare). Glycoproteins precipitated by KM694 were revealed by direct ECL Plus detection. Luminescence was captured using an Image Master VDS-CL box imager (Amersham Pharmacia Biotech).

### Mass spectrometry analysis

For mass spectrometry identification of glycoproteins purified by DBA lectin affinity, proteins (1 mg) from a mix of follicular fluid of large and small follicles (one mix for each genotype) were incubated with 60 µL agarose-coupled DBA lectin in RIPA buffer, as indicated above. After the last wash step, purified glycoproteins were eluted from the precipitated complex through incubation with 0.2 M of N-Acetyl-D-galactosamine (Sigma) for 90 min at room temperature under stirring. After centrifugation (10000 g, 2 min), the supernatant was collected and frozen for subsequent analysis. Eluted samples were submitted to SDS-PAGE in 10% polyacrylamide gels (12 min at 90 V) and stained by Coomassie blue. Proteins were in-gel digested with trypsin as previously described [68]. Each peptide mixture from +/+ and L/L genotype was analyzed in triplicate by nanoflow liquid chromatography tandem mass spectrometry (nanoLC-MS/MS). All experiments were performed on a LTQ Orbitrap Velos Mass Spectrometer (Thermo Fisher Scientific, Bremen, Germany) coupled to an Ultimate 3000 RSLC chromatographer (Dionex, Amsterdam, The Netherlands). Samples were loaded on an LCPackings trap column (Acclaim PepMap 100 C18, 100 µm i.d×2 cm long, 3 µm particles) and desalted for 10 min at 5 µL/min with 4% solvent B. Mobile phases consisted of (A) 0.1% formic acid, 97.9% water, 2% acetonitrile (v/v/v) and (B) 0.1% formic acid, 15.9% water, 84% acetonitrile (v/v/v). Separation was conducted using a LCPackings nano-column (Acclaim PepMap C18, 75 µm i.d×25 cm long, 3 µm particles) at 300 nl/min by applying gradient consisted of 4–45% B for 120 min. The mass spectrometer was operated in data dependent scan mode. Survey full scan MS spectra (from 300–1800 m/z) were acquired in the Orbitrap analyser with R = 30 000. The 20 most intense ions were fragmented in the high-pressure linear ion trap by collision-induced dissociation. Dynamic exclusion was active during 30 s with a repeat count of 1. Polydimethylcyclosiloxane (*m/z*, 445.1200025) ions were used for internal calibration.

MS/MS ion searches were performed using Mascot search engine v 2.2 (Matrix Science, London, UK) against the mammalian section of Uniprot_sprot database (2012_07). The search parameters included trypsin as a protease with allowed two missed cleavages, carbamidomethylcysteine, methionine oxidation and acetylation of N-term protein as variable modifications. The tolerance of the ions was set to 10 ppm for parent and 0.8 Da for fragment ion matches. Mascot results obtained from the target and decoy databases searches were subjected to Scaffold 3 software (v 3.4.3, Proteome Software, Portland, USA). The raw data may be downloaded from ProteomeCommons.org linked to the Tranche data repository using the “follicular_fluid_sheep” keywords. Peptide and protein identification was done by the Peptide and Protein Prophet algorithms [69], [70] with a probability >95.0%. Only proteins with greater than four identified peptides were considered. Differentially expressed proteins were determined using the spectral counting quantitative module of Scaffold 3 Q+ software (version 3.4, Proteome Software, Portland, USA). To eliminate quantitative ambiguity into protein groups, we ignored all the spectra matching any peptide that is shared between proteins. Thereby, quantification performed with normalized spectral counts was carried out on distinct proteins identified from two biological replicates. T-test was performed to differentiate the significantly changed proteins with a p-value <0.05 between the two genotypes.

### Data analysis

All experimental data are presented as means ± SEM. The genotype effect on gene expression was analyzed using t-test for comparisons between two means. For all analyses, differences with P>0.05 were considered as not significant.

## Supporting Information

Figure S1 Expression of genes within the “one recombinant” interval of the *FecL* locus. One µg of total RNA from granulosa cells (GC) and theca cells (TC) from large (LF, ≥6 mm) follicles, pituitary gland (PG) and hypothalamus (HPT) were reverse-transcribed and submitted to real-time PCR analysis for quantification of *PHOSPHO1, ABI3, GNGT2, SNF8, UBE2Z, ATP5G1, CALCOCO2* gene expression. Total RNA from intestine were reverse-transcribed and submitted to real-time PCR analysis for quantification of *GIP* and *B4GALNT2* gene expression. Data are means ± SEM of relative expression to the reference gene *RPL19* showed on a log scale. No significant difference was found between means (n = 5) from non-carriers (*+/+*) and homozygous carriers of the *FecL^L^* mutation (*L/L*).
(TIFF)

Figure S2 Expression of IGF2BP1 target genes in Lacaune sheep granulosa cells. A. One µg of total RNA from granulosa cells (GC) from small (SF, 1–3 mm), and large (LF, ≥6 mm) follicles were reverse-transcribed and submitted to real-time PCR analysis for quantification of *MYC* gene expression. Data are means ± SEM of relative expression to the reference gene *RPL19* showed on a log scale. Asterisk indicates a significant difference between means (n = 5) from small and large follicles of the same genotype. ***: p<0.001. No significant difference was found between *+/+* and *L/L* genotypes. B. Twenty-five µg of granulosa cell protein extracts from +/+ and L/L large antral follicles were separated on SDS-PAGE, transferred on nitrocellulose membrane and revealed by immunoblotting using ß-actine rabbit polyclonal antibody (1/1000). No significant difference was found between *+/+* and *L/L* genotypes.
(TIFF)
