# Supplementary figures and images for: The Highly Prolific Phenotype of Lacaune Sheep Is Associated with an Ectopic Expression of the B4GALNT2 Gene within the Ovary
Source: PLoS Genet. 2013 Sep 26;9(9):e1003809. doi: 10.1371/journal.pgen.1003809 (PMC3784507; doi:10.1371/journal.pgen.1003809)

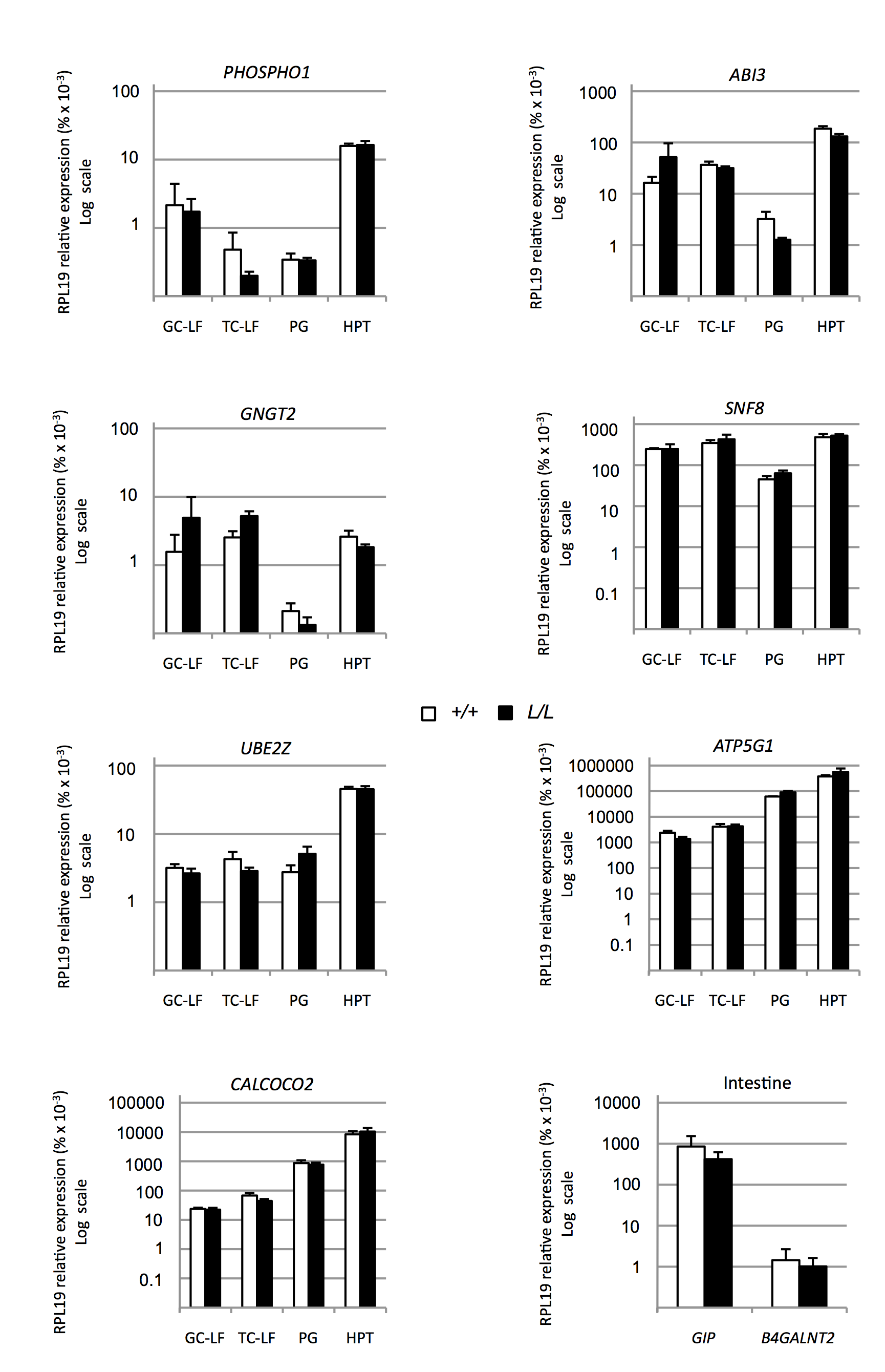

Supplement: Figure S1 — Expression of genes within the “one recombinant” interval of the FecL locus. One µg of total RNA from granulosa cells (GC) and theca cells (TC) from large (LF, ≥6 mm) follicles, pituitary gland (PG) and hypothalamus (HPT) were reverse-transcribed and submitted to real-time PCR analysis for quantification of PHOSPHO1, ABI3, GNGT2, SNF8, UBE2Z, ATP5G1, CALCOCO2 gene expression. Total RNA from intestine were reverse-transcribed and submitted to real-time PCR analysis for quantification of GIP and B4GALNT2 gene expression. Data are means ± SEM of relative expression to the reference gene RPL19 showed on a log scale. No significant difference was found between means (n = 5) from non-carriers (+/+) and homozygous carriers of the FecLL mutation (L/L). (TIFF) [file pgen.1003809.s001.tiff]

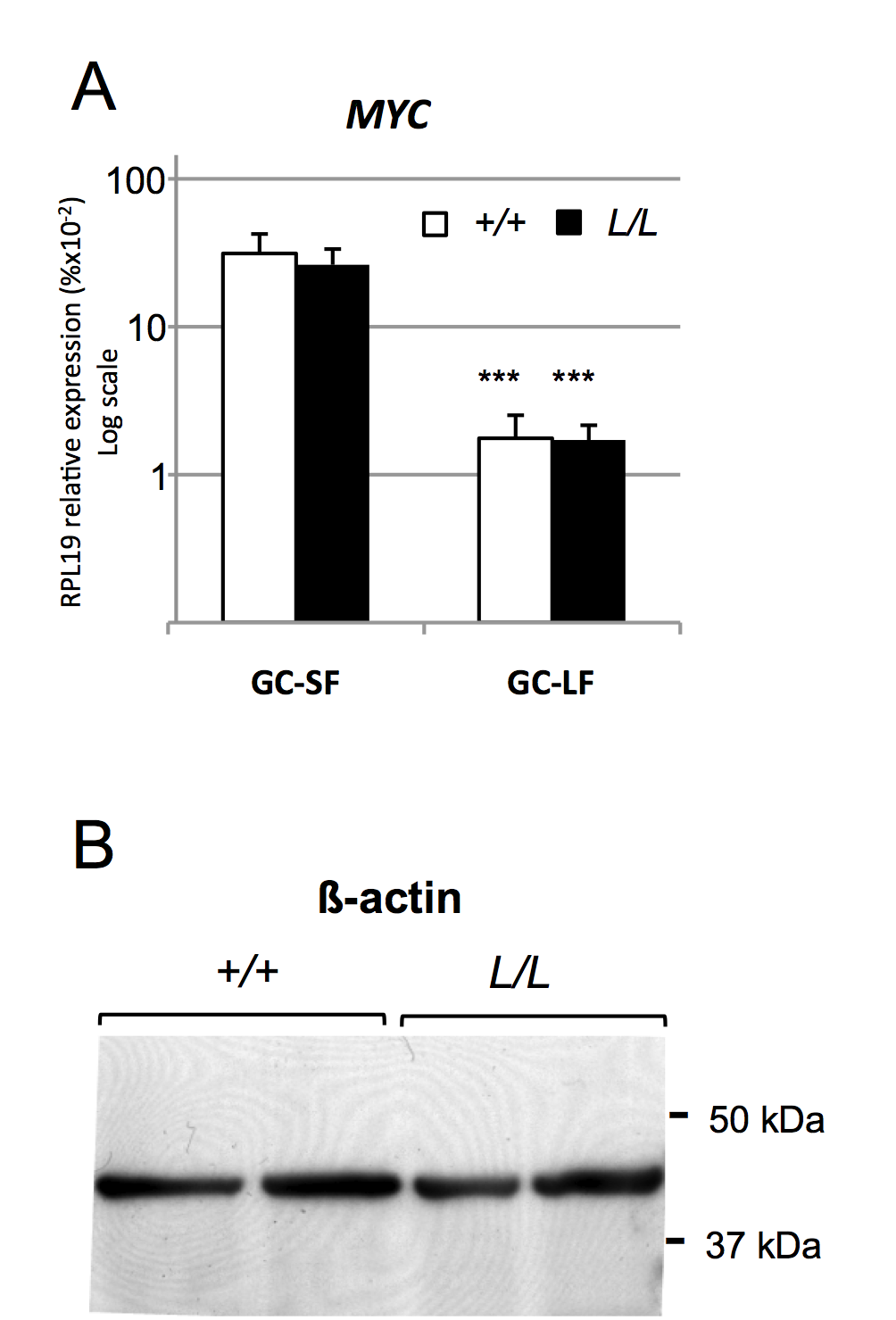

Supplement: Figure S2 — Expression of IGF2BP1 target genes in Lacaune sheep granulosa cells. A. One µg of total RNA from granulosa cells (GC) from small (SF, 1–3 mm), and large (LF, ≥6 mm) follicles were reverse-transcribed and submitted to real-time PCR analysis for quantification of MYC gene expression. Data are means ± SEM of relative expression to the reference gene RPL19 showed on a log scale. Asterisk indicates a significant difference between means (n = 5) from small and large follicles of the same genotype. ***: p<0.001. No significant difference was found between +/+ and L/L genotypes. B. Twenty-five µg of granulosa cell protein extracts from +/+ and L/L large antral follicles were separated on SDS-PAGE, transferred on nitrocellulose membrane and revealed by immunoblotting using ß-actine rabbit polyclonal antibody (1/1000). No significant difference was found between +/+ and L/L genotypes. (TIFF) [file pgen.1003809.s002.tiff]
